# Potential Crosstalk between Fructose and Melatonin: A New Role of Melatonin—Inhibiting the Metabolic Effects of Fructose

**DOI:** 10.1155/2018/7515767

**Published:** 2018-08-01

**Authors:** Francisco J. Valenzuela-Melgarejo, Claudia Caro-Díaz, Gerardo Cabello-Guzmán

**Affiliations:** Laboratory of Molecular Cell Biology, Department of Basic Sciences, Universidad del Bío-Bío, Campus Fernando May, Chillán, Chile

## Abstract

Increased consumption of energy-dense foods such as fructose-rich syrups represents one of the significant, growing concerns related to the alarming trend of overweight, obesity, and metabolic disorders worldwide. Metabolic pathways affected by fructose involve genes related to lipogenesis/lipolysis, beta-oxidation, mitochondrial biogenesis, gluconeogenesis, oxidative phosphorylation pathways, or altering of circadian production of insulin and leptin. Moreover, fructose can be a risk factor during pregnancy elevating the risk of preterm delivery, hypertension, and metabolic impairment of the mother and fetus. Melatonin is a chronobiotic and homeostatic hormone that can modulate the harmful effects of fructose via clock gene expression and metabolic pathways, modulating the expression of PPAR*γ*, SREBF-1 (SREBP-1), hormone-sensitive lipase, C/EBP-*α* genes, NRF-1, PGC1*α*, and uncoupling protein-1. Moreover, this hormone has the capacity in the rat of reverting the harmful effects of fructose, increasing the body weight and weight ratio of the liver, and increasing the body weight and restoring the glycemia from mothers exposed to fructose. The aim of this review is to show the potential crosstalk between fructose and melatonin and their potential role during pregnancy.

## 1. Introduction

The cooccurrence of several metabolic problems affecting the population has been described, including obesity, hyperglycemia, dyslipidemia, and hypertension, all of which lead and also increase the risk of death [1]. Increased consumption of energy-dense foods and sugar-sweetened beverage represents one of the significant, growing concerns related to the alarming trend of overweight, obesity, and metabolic disorders worldwide. There are currently various sugars that are added to foods as supplements to improve the commercial properties of some products; among them are sucrose, fructose, and honey [2]. However, there has been an increase in the consumption of fructose-rich syrups for 50 years and it today oscillates around 40% of daily sweetener consumption [2]; this is due to the high sweetening power of fructose over other available sugars such as glucose [2]. Fructose-rich syrups are derived from maize (fructose 42–55%) and exhibit a strong sweetening power, ranging from 1.16–1.28 times compared to glucose (0.76) [2], which explains the widespread use of this sugar in the food industry. Fructose consumption has gone from an average intake of 8.1 kg/person in the 90s to a consumption of 65 kg/person per year during 2016 [3], and probably, these analyses may underestimate the actual fructose consumption [4], which could represent 18% higher than the estimated to date [5].

Fructose was considered an ideal sugar to supplement foods, due to its low glycemic index in function of its rapid incorporation to glycolysis and the bypass that makes on the activity of hexokinase, allowing its rapid metabolic incorporation. However, it has been observed that excessive fructose consumption increased the risk of insulin resistance and lowering postprandial peak blood glucose level. Also it induces a high circulating level of low-density lipoprotein (LDL), elevated triglycerides, and increased risk of developing obesity [3, 6, 7]. If this consumption of fructose is with excessive intake of fats, the rise of resistance to leptin hormone, hypertension, and cardiovascular disease will happen [8, 9]. Finally, several of these alterations lead to the appearance of metabolic syndrome, enhanced with the possible intracellular glucocorticoid production of adipose tissue [9].

## 2. Fructose and Metabolism

Fructose is a ketohexose that can be present in many fruits via hydrolysis of sucrose; uptake in the intestine occurs via several glucose transporters that had the capacity to a greater or lesser degree to transport the fructose such as transporters Sglt1 and glucose transporter-5 (GLUT-5) [2]. However, during the year 2009, employing confocal microscopy in a mouse intestine, dense labeling of Glut-5 in the hairy portion of the jejunum was observed suggesting that this transporter could be the main protagonist of fructose transport [10]. With this observation plus a series of radioactive labeling experiments of C^14^-labeled sugars, 75% of the absorbed fructose occurs through the GLUT-5 transporter [10]. GLUT-5 is expressed not only in the intestine [11] but also in other cell types such as skeletal muscle [12, 13], kidney [11], adipose tissue [14], and pneumocyte [15], suggesting the ubiquitous ability of fructose uptake in the body. The highest percentage of fructose from fructose-containing food passes into the bloodstream through the transport of fructose/glucose via GLUT-2 transporter in the basal side of the enterocyte [16], following an increase in the portal circulation of fructose approximately twofold (0.025 to 0.05 mM) and finally producing an increase of about four times at a systemic level [17].

**Figure 1:**
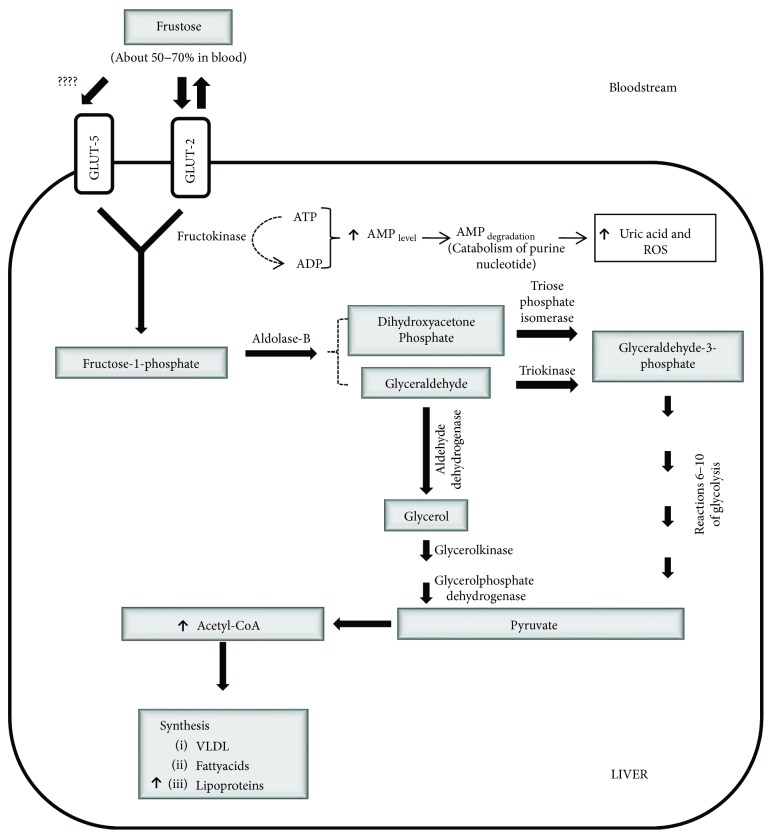
Metabolization of fructose in the liver cell. The main transporter of fructose in the liver is Glut-2, which translocates ketohexose to the cytoplasm and is transformed by the enzyme fructokinase to fructose-1-phosphate. After that, it is split into dihydroxyacetone phosphate and glyceraldehyde (triose phosphates). The final step is the production of pyruvate and their transformation to acetyl-CoA by pyruvate dehydrogenase; the latter product is a critical substrate for de novo lipogenesis.

Uptake of about 50–70% of the circulating fructose is made by the liver [17] suggesting that the dominant player in the metabolism of fructose is hepatocyte. Fructose is uptake by the liver via GLUT-2 [18] or to a lesser degree, given of GLUT-5 [19]. Hepatic fructose is converted to glyceraldehyde and dihydroxyacetone, and both molecules can be transformed to glyceraldehyde-3-phosphate and advanced via glycolysis to pyruvate (inhibiting glycolysis) and converted via dihydroxyacetone into glycerol-3-phosphate and finally into acetyl-CoA and lead to the synthesis of VLDL fatty acids and lipoproteins [20] (see Figure 1). At the level of peripheral tissues, such as muscle, uptake of fructose can be made via GLUT-5 [12, 13]. However, muscle cell does not express fructokinase, and the high value of Km of hexokinase for fructose suggests that this pathway is not critical for fructose metabolism [21].

Curiously, fructose supplementation of foods induces a higher absorption of fructose (positive feedback), through an increase in the transport of fructose by GLUT-5 of the enterocyte and by inducing the transcriptional activation of the GLUT-5 gene in about seven times [10]. The positive regulation of fructose uptake by GLUT-5 also can be induced by glyceraldehyde supplementation [16] indicating that GLUT-5 expression not only is dependent on its ligand but also is a product of fructose metabolism.

## 3. Consequences of Excessive Consumption of Fructose: A New Risk Factor during Pregnancy

At a molecular level, chronic exposition to fructose induce an upregulation of sterol regulatory element binding protein-1 (SREBP-1c) and the enzyme acetyl-coenzyme A carboxylase that can lead to increase of fat synthesis and hyperuricemia by high degradation of AMP and depletion of ATP content in the liver, all caused by the excessive synthesis of fructose-1-phosphate, a consequence of the excessive incorporation of fructose into blood circulation [8] which adds to liver inhibitions of NRF-2 (antioxidant transcription factor and liver steatosis inhibitor) and PGC1*α* expression [22] and stimulation of gluconeogenesis via FOXO1 and PEPCK expression [23]. All of which can also produce an increased risk of insulin resistance, a high circulating level of LDL, elevated triglycerides, increased risk of developing obesity [3], and rising resistance to leptin [8]. Similarly, the acute consumption of fructose in obese subjects showed an elevation of weight and liver fat, stimulation of de novo lipogenesis, insulin secretion, and decreased hepatic *β*-oxidation [24].

The absorption of fructose in the intestine occurs in parallel with the uptake of NaCl, and curiously, an increase of absorption in about 25% is detected in the presence of fructose. The final output of that is an increase in body volume due to the sodium hydration cloud, resulting in a significant rise in systolic pressure by approximately 10% [10] and finally hypertension [8, 25]. During pregnancy, fructose consumption may increase the risk of preterm delivery in an animal model [3] and can be associated with high levels of homocysteine [26], suggesting a possible vascular alteration associated to hypertension and, if we extrapolate to human pregnancy, a possible preeclampsia [27]. Moreover, fructose consumption during pregnancy increases the plasmatic uric acid level [28] and the body-mass-and-liver ratio. Besides, fructose increases the circulating levels of glucose, insulin, free fatty acids, triglycerides, cholesterol, lipase, insulin, TNF*α*, and IL-*β*. In the liver, fructose induces hepatic steatosis by overexpression of the SREBP-1c and FASN and a decrease in the expression of PGC1*α*, PPAR*α*, and phosphoenolpyruvate carboxykinase [26, 29]. Also in the liver, fructose induces mRNA expression from Glut-2, Glut-5, fatty acid synthase (FAS), and acetyl-CoA carboxylase (ACC1) [29]. Those metabolic symptoms are more severe in ovariectomized animals where the triglyceride levels are three times higher than the control diet. Effects partially reverted in ovariectomized animals supplemented daily with estradiol [30], suggesting that estradiol can protect against the adverse effects of fructose [31] or eventually hypertension [30].

Fructose also increases in about 2-fold the insulin levels in term-pregnant rats, raising plasma levels of fructose in embryos and newborns by about 1200 and 600 times, respectively. Moreover, fructose decreases placental mass by about 10% [8], inhibits the placental expression of NRF-2 and heme oxygenase-1 (HO-1), and induces oxidative stress and a high level of lipid peroxidation in the liver of fetuses [28]. Additionally, during labor, fructose consumption has been shown to induce increased pyruvate and lactate content in newborns and increases maternal acidosis, elevating the risk of developing complications during labor. At the same time, increased glycemia, stimulation of gluconeogenic enzymes, triglyceridemia, high levels of expression of enzymes ACC2 and carnitine palmitoyltransferase (CPT1a), decreased antioxidant enzymes and promoter cofactors of *β*-oxidation-like PGC1*α* (major regulator of mitochondrial biogenesis) and PPAR*α* [8], and acetyl-coenzyme A carboxylase [8] are observed. Similarly, in newborns at 3 weeks old, an increase in body weight, blood glucose, insulin blood, and liver triglyceride [32] is detected and later (at 9–16 weeks of age), an increase in systolic blood pressure [33, 34] and hypoglycemia and an increase in the liver expression of phosphoenolpyruvate carboxykinase are observed [26]. The several adverse effects of fructose described during fetal/neonatal life suggest to fructose is a high-risk factor during the pregnancy.

## 4. A New Role of Melatonin: Inhibiting the Metabolic Effects of Fructose

### 4.1. Melatonin and Metabolism

Melatonin is a lipophilic indoleamine hormone synthesized by the pineal gland that shows a plasmatic peak during dark hours that gives chronobiologic information for the circadian organization of metabolism. A second function proposed for melatonin is a homeostatic role, regulating several aspects of fetal physiology such as the development and maintenance of fetal physiology or pregnancy. The third function of melatonin is to modulate the redox status, via scavenger activity or regulating the expression of antioxidant enzymes [35]. Melatonin acts through membrane receptors named MT1 and MT2, and both are G protein-coupled receptors; MT1 is associated with Gi protein and inhibition of adenylyl cyclase and stimulation of Ca^2+^-phospholipase C, whereas the MT2 receptor is associated with protein kinase C stimulation and increase of Ca^2+^-IP_3_ [35]. Curiously, the concentration of melatonin in mitochondria is higher than blood, and this elevated concentration can be induced by PEPT1-2 transporter or the tissue-specific endogenous synthesis of melatonin by mitochondria [36, 37].

The inner membrane of mitochondria is the site of the final step in electron transport of oxidative phosphorylation and is the site of reduction of O_2_ to water by a cytochrome c oxidase (complex IV), but a dissipation of electrons by complex I and III and generation of incomplete reduced oxygen species such as superoxide, hydrogen peroxide, and hydroxyl radical are occasionally observed [36–38]. The elevated concentration of melatonin observed in mitochondria can be helping in antioxidant protection, due to its free radical scavenger capacity [36, 37] or the potential signal transduction and modulation of complex I activity, generated by the MT1 or MT2 receptors expressed in the inner membrane of the mitochondria [37, 39]. Also, the expression of antioxidant enzymes such as GPx, GRd, SOD, and iNOS [38, 40, 41] can be modulated by melatonin, giving the capacity for antioxidant protection from carcinogen substances such as safrole, Fenton's reagents, glutathione depletion, carbon tetrachloride, and ionizing radiation and reduces the toxicity of cyanide and hydroperoxide production [38, 42]. Moreover, melatonin inhibits mitochondrial permeability transition pore opening and the apoptosis induced by cytochrome c release [40, 41, 43].

Mitochondria are a critical protagonist of cell metabolism and bioenergetics pathways such as fatty acid *β*-oxidation, pyruvate oxidation, citric acid oxidation, and amino acid oxidation together with the protein carriers of oxidative phosphorylation (inner membrane). Curiously, mitochondrial dysfunction observed in obesity, diabetes, metabolic syndrome, and dyslipidemias showed a reduction of mitochondria number after high-fat feeding [40], reduction of mitochondrial size, and mitochondrial fission in diabetic mice [44]. Melatonin has the capacity to increase the activity of mitochondrial complex I and IV, elevate the synthesis of ATP in a dose-dependent manner [36], revert the progressive reduction of mitochondrial oxygen consumption during sepsis [45], and restore the GSH, GPx, and GRd activities in mitochondria after redox insult during sepsis [46].

At the supraphysiological level, the circadian production of melatonin by the pineal gland gives the temporal signal for the metabolic organization of glucose, lipid, and adiposity [47]. Studies in the suprachiasmatic nucleus of the hypothalamus have shown that the uptake of ^14^C-deoxyglucose is almost imperceptible during night hours but the pattern of glucose uptake reaches a maximum during light hours, which allows the 24 h oscillation of cell transcriptome [48]. At the molecular level, circadian rhythms are governed by a transcriptional/translational circuit of genes called clock genes, which are named Bmal-1, Per-1–3, Clock, Cry1-2, and the modulating factors PGC1*α* and Rev-erb*α* [1]. The clock genes Bmal-1/Clock can be linked to consensus sequence sites on promoters from Per and Cry genes or over promoters of genes controlled by Clock such as hexokinase [35] or regulate the expression of a cofactor and coregulator such as histone acetyltransferase p300/CBP. All of which determine the circulating levels of hormones related to metabolisms such as insulin, glucagon, growth hormone, glucocorticoid, and thyrotropin [48]. Impaired circadian rhythms by nocturnal exposure to light and suppression of the hormone melatonin are known as “chrono-disruptions” and have been associated with metabolic diseases such as increased risk of type 2 diabetes, increased weight gain, hypertrophy of adipose tissue, gestational diabetes, and dyslipidemia [1, 48, 49]. Moreover, the total inhibition of circadian clock by knockdown of clock genes results in inhibition of insulin response and ectopic accumulation of fat. Besides the decrease in numbers of mitochondria, downregulation of oxidative phosphorylation and lowering of the expression of GLUT-4, hexokinase type 2 (HK-2), and phosphofructokinase and lowering of postprandial glucose uptake [50] and decreased activity of pyruvate dehydrogenase [49] and hypoglycemia due to the uncoordinated expression of genes that regulate glycogenolysis are observed [49].

A rat exposed to melatonin reestablishes the diurnal locomotor activity circadian rhythms, glycemia, and insulinemia inhibited by diet-induced obesity [51]. Moreover, the nocturnal administration of melatonin reduces the insulin production, food intake, and body weight gain [52]. Similarly, male rat feeding with diet-induced obesity and exposure to melatonin have been observed with a reduction in body mass and visceral adiposity [53]. Moreover, the combination of melatonin and metformin produces a synergistic effect on body mass, suggesting that melatonin prevents the metabolic disease [51]. At the molecular level, melatonin can increase the expression and activity, via a melatonin receptor, of SIRT1 and inhibit the acetylation of PGC-1*α* after cadmium treatment in hepatocyte culture [54], suggesting the ubiquitous effects of melatonin. However, different reports talk in detail how melatonin could modify the metabolism related to lipogenesis and lipolysis in the animal model. For example, treatment with high concentration of melatonin (1 mM) in the adipose cell (crucial in the development of metabolic syndrome) induces the differentiation and the formation of small lipid droplets and mitochondria. Moreover, it enhances the expression of PPAR*γ* and C/EBP-*α* genes and induces the lipolysis similar to that induced by isoproterenol via stimulation of expression of hormone-sensitive lipase (HSL), NRF-1 (nuclear respiratory factor-1), PGC1*α*, and uncoupling protein 1 (UCP-1)—a marker of brown adipose tissue [55]. Similarly, melatonin in oocytes induces the increase of amounts of lipid droplets and mitochondria, reduces the size of lipid droplets, and stimulates the lipogenic, lipolytic, and *β*-oxidation pathways inducing the expression of PPAR*γ*, SREBF-1 (SREBP-1), PGC1*α*, and hormone-sensitive lipase [56]. In contrast, the brown adipose tissue (BAT) from newborn showed an induction of the expression PGC-1*α*, UCP-1, PPAR*α*, PPAR*γ*, and C/EBP-*α* genes in animals with mothers exposed to constant light (inhibitor of melatonin production) [57].

Curiously, subjects with metabolic syndrome and type 2 diabetes, are detected to have a melatonin/insulin ratio lower than lean people, 9.64 for subjects with metabolic syndrome and type 2 diabetes and 15.36 for lean people [58], and obese nondiabetic subjects showed a night melatonin concentration higher than those of lean patients and type 2 diabetes patients [59]. Moreover, in rat treated 15 days with melatonin, restoration of the specific activities of hepatic hexokinase, glucokinase, and glucose 6-P dehydrogenase from streptozocin-diabetic rats [60] is observed, and treatment with melatonin has the capacity of stimulating SIRT3 activity which deacetylates other metabolic genes such as FOXO3A, SOD2, and tricarboxylic acid enzymes [61]. Besides, melatonin suppressed the Warburg metabolisms of cancer, via the inhibition of glucose uptake, lactate, and LDH activity and increase in glycogen reservoirs in sarcoma cells [62]. Similarly, in rat Sertoli cells, melatonin decreased LDH protein levels, lactate production, and alanine production and induced acetate content (essential for the maintenance of a high rate of lipid synthesis) [63]. Besides, it restores the mitochondrial function over metabolism, plays a role in deciding stem cell fate of adipose-derived stem cells, and inhibits adipogenic differentiation via inhibition of expression of PPAR*γ*, C/EBP*α*, and lipoprotein lipase [64, 65]. However, melatonin is critical for circadian expression of PPAR*γ* in mature adipose tissue [66], suggesting temporal and tissue-specific effects of melatonin during differentiation.

### 4.2. Fructose and Chronobiologic Metabolism

At the circadian level, excess fructose at inadequate hours has been found to alter the individual's metabolic and circadian response, as observed in mice fed with fructose during daylight hours, where there is an increase in levels of insulin and leptin by about 50%, compared to mice fed with fructose during dark hours [67]. At the same time, mice feeding during the day or night hours for six weeks induce an increase of body weight in about 1.8 and 1.3 times higher, respectively, than ad libitum [67]. At the liver level, fructose inhibits the circadian rhythm of Bmal-1 and causes a delay in the peak expression of Per-1 and Clock in about 9 and 2 hours, respectively. At the same time, it reduces the amplitude of Bmal-1 and Per-1 expression and decreases the rate of phosphorylation of pAMPK and p-ACC, critical enzymes in the inhibition of translocation of fatty acids to mitochondria, and its subsequent *β*-oxidation, finally inducing the synthesis of lipids in the liver [68]. At the muscle level, fructose induces an increase in the amplitude of oscillation of the Per-1 and Bmal-1 genes and an increase in the phosphorylation ratio of pAMPK, p-ACC, and the expression of transcription factor PPAR*α* (stimulator of *β*-oxidation) which results in an inhibition of translocation of fatty acids to the mitochondria and thereby increasing the availability of substrate for *β*-oxidation [68]. This previous metabolic desynchronization adds the observation in patients treated with fructose-sweetened beverages, where the area under the curve during 24 hours (AUC) of plasma glucose and insulin levels is lower than those of patients who drank glucose-sweetened beverages. Concurrently, these fructose-sweetened beverages induce an increase in triglyceride production (AUC), indicating a potential correlation between the circadian system and fat metabolism modified by fructose [69].

Melatonin is a chronobiologic and homeostatic agent in different physiological systems [35], able to inhibit the expression of clock genes, as it happens with the inhibition of Bmal-1 and Per-2 in the adrenal gland [70, 71]. Thus, it can modify the circadian functions of different physiological systems. In experiments performed with mice of 6 weeks feeding with fructose plus melatonin, melatonin reverted the effects of visceral fat accumulation, the increase in leptin and uric acid and insulin resistance, and the increase in blood pressure by fructose treatment with melatonin [72, 73]. Also, melatonin has been shown to have a vasodilatory effect on the cerebral arteries of sheep fetuses [74] which has been reverted in the newborn whose mothers were feeding with fructose-rich diet, reverting hypertension and increased renal expression of the soluble epoxide hydrolase enzyme (correlated with hypercholesterolemia) as well [75].

**Figure 2:**
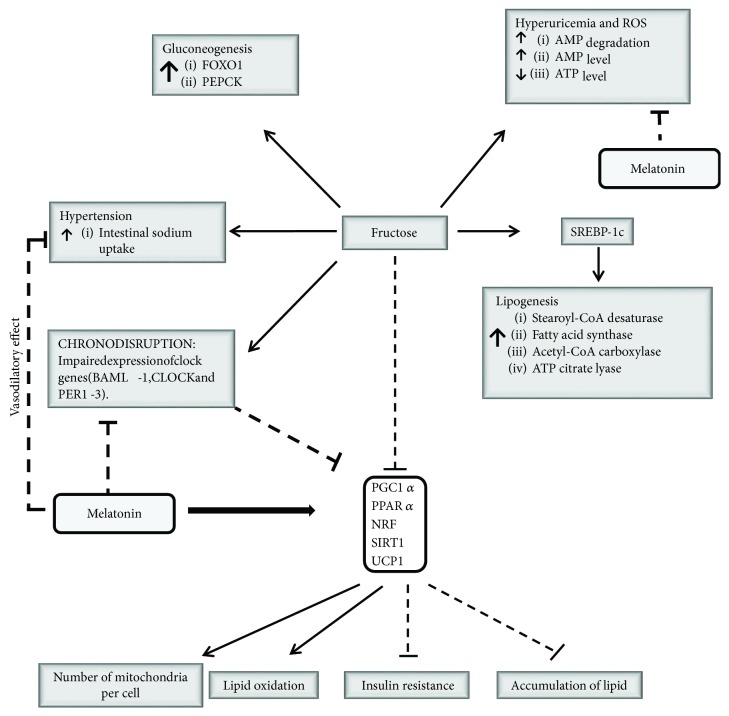
Potential crosstalk between melatonin hormone and fructose. Fructose induces lipogenesis via SREBP-1c stimulation [26, 29], hypertension [8, 10, 25–27, 33, 34], gluconeogenesis [23, 29], hyperuricemia, and reactive oxygen species (ROS) [8, 28]. Besides, it induces chrono-disruption [67, 68], and the impairment expression of clock genes modifies the circadian output of PGC1*α*, PPAR *α*, NRF, SIRT1, and UCP1 [1, 48–50]. The molecular inhibition of PGC1*α*, PPAR *α*, NRF, SIRT1, and UCP1 by fructose [8, 22, 26, 28, 29, 32, 82, 83] can be reverted by melatonin exposition [44, 51, 54–56]. Similarly, melatonin reverted the chrono-disruption, hyperuricemia, hypertension, and impaired expression of clock genes [72–74, 84–86], finally modulating the negative effects of fructose on metabolism.

**Figure 3:**
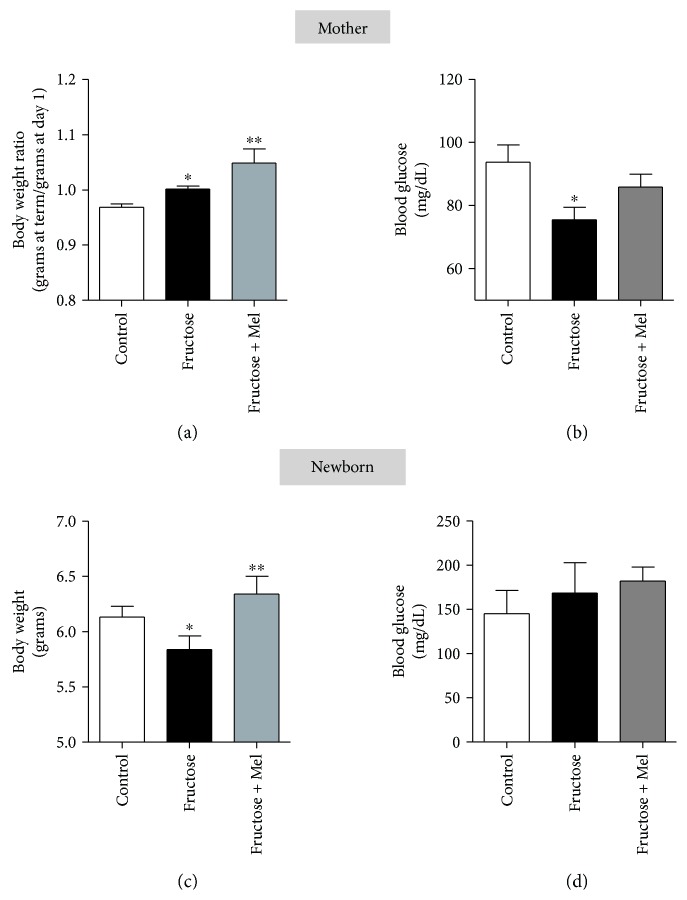
Effects of fructose and fructose plus melatonin over mothers and newborns in body weight and blood glucose levels. (a and b) show the body weight ratio (a) at term compared to the first day of pregnancy and blood glucose level (b) from 4 mothers maintained with standard diet (control) or supplemented daily with fructose (*n* = 4) or fructose plus melatonin (fructose + Mel, *n* = 4)). (c and d) show the body weight (c) and blood glucose level (d) from newborns (3–5 days). Wistar rats (200–250 g) were fed daily with standard diet (pellet) or supplemented with fructose-rich syrups equivalent to 2.7 g/kg/day (Great Value, USA) or fructose plus melatonin (Sigma-Aldrich, USA). Melatonin supplementation was given daily in drinking water at 0.1 mg/kg/day beginning at the third week and maintained after mating. Measurement of body weight and glycemia was performed from newborns euthanized by decapitation. We obtained the blood sampling in the tail tip, previously gently massaging the tail, and then using the Accu-Chek test strip system for glycemia measurement. The weight of the mothers as expressed with the ratio between body weights at term versus the first day after mating (grams at term/grams at day 1). ^∗^Different from control, *P* < 0.05, one-way ANOVA, Newman–Keuls posttest. ^∗∗^Fructose versus fructose + melatonin. The protocol was approved by the Ethics Committee of the University of Bío-Bío.

Due to the several effects of melatonin on metabolism and the potential crosstalk with fructose (see Figure 2), we speculate that melatonin can modulate the adverse effects of fructose; for this purpose, we maintained pregnant rats with standard diet, high-fructose diet alone, or high-fructose diet plus melatonin. The mothers exposed to fructose induce an increase in body weight similar to that observed previously by Yuruk and Nergiz-Unal [32], and for the first time, we observed an enhanced effect of fructose by melatonin (Figure 3(a)), similar with the increase in body weight by melatonin observed previously in patients with mood disorder treated with antipsychotics [76]. Moreover, we detected in mothers, exposed to fructose, a low level of blood glucose (Figure 3(b)), different from those reported previously [32]. We speculate that these differences are due to the low level of fructose intake of our experiment design (2.7 g/kg/day) or the diet with a low level of fructose may help control glycemic index similar to that reported previously for fructose [77–79].

**Figure 4:**
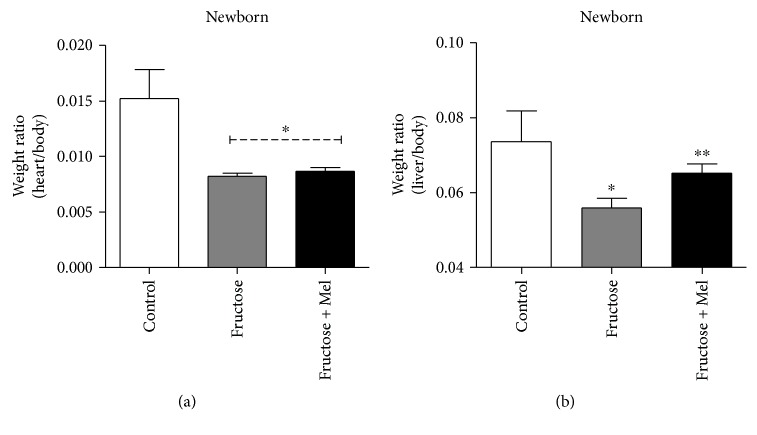
Effects of fructose and fructose plus melatonin in the heart/body weight ratio (a) and liver/body weight ratio (b) of the newborns (3–5 days), from mothers fed daily with standard diet (control) or supplemented with fructose-rich syrups or fructose plus melatonin. ^∗^Different from control, *P* < 0.05, one-way ANOVA, Newman–Keuls posttest. ^∗∗^Fructose versus fructose + melatonin. The protocol was approved by the Ethics Committee of the University of Bío-Bío.

The newborn rat exposed during the pregnancy to fructose showed a reduction of body weight in about of 5% than control (Figure 3(c)); this observation is similar to that reported by Asghar et al., in pregnant mice [80]. During pregnancy, the melatonin supplementation to mothers exposed to fructose recovered the body weight of the newborn (Figure 3(c)); we do not detect a difference in blood glucose levels (Figure 3(d)), biparietal diameter, and femur length in newborns in different treatments (data not showed). Moreover, the treatment with fructose reduces the weight ratio of the heart and body and melatonin has no effects on the heart mass (Figure 4(a)). However, the weight ratio of the liver and body showed a reduction with fructose treatment during pregnancy; effects are reverted by melatonin supplementation (Figure 4(b)), similar to those observed in liver injuries by fluoride and treated previously with melatonin [81].

## 5. Conclusion

Several metabolic points modified by fructose can be modulated by melatonin, via chronobiologic and homeostatic actions, suggesting crosstalk between fructose and melatonin hormone. These melatonin effects on fetuses and newborns exposed to fructose during pregnancy have not yet been studied at the molecular level. However, the available data suggest the adverse effects of fructose in pregnancy which can be reversed partially by melatonin; this can be due to the capacity of the pineal hormone to modify the expression of metabolic genes such as PGC1*α*, PPAR*α*, PPAR*γ*, NRF, SIRT1, and C/EBP*α*, which are targets of fructose. Therefore, melatonin can play a role in adult life and pregnancy, partially protecting against metabolic syndrome induced by fructose.

## Glossary

PPAR: Peroxisome proliferator-activated receptor
NRF-1: Nuclear respiratory factor-1
C/EBP: CCAAT/enhancer-binding protein
GLUT-5: Glucose transporter-5
GLUT-2: Glucose transporter-2
UCP-1: Uncoupling protein 1
SREBP-1: Sterol regulatory element-binding protein 1 (stimulator of transcription of sterol-regulated genes)
PGC1*α*: Peroxisome proliferator-activated receptor-gamma coactivator 1 alpha
FASN: Fatty acid synthase
PEPCK: Phosphoenolpyruvate carboxykinase
ACC1: Acetyl-CoA carboxylase
CPT1a: Carnitine palmitoyltransferase 1a
AMPK: 5-Prime-AMP-activated protein kinase
LDL: Low-density lipoprotein
SIRT: Sirtuin
Bmal-1: Aryl hydrocarbon receptor nuclear translocator-like protein 1
Clock: Circadian locomotor output cycles kaput
Per: Homolog of period, drosophila
E-Box: Promoter sequence for binding of clock-Bmal-1 complex (CACGTG)
ROS: Reactive oxygen species.
